# Shifting power: data democracy in engineering solutions

**DOI:** 10.1088/1748-9326/ad7614

**Published:** 2024-09-17

**Authors:** Bethany B Cutts, Uchenna Osia, Laura A Bray, Angela R Harris, Hanna C Long, Hannah Goins, Sallie McLean, Jacqueline MacDonald Gibson, Tal Ben-Horin, Astrid Schnetzer

**Affiliations:** 1 Department of Parks, Recreation and Tourism Management, NC State University, Raleigh, NC, United States of America; 2 Center for Geospatial Analytics, NC State University, Raleigh, NC, United States of America; 3 North Carolina Center for Coastal Algae, People and Environment, Raleigh, NC, United States of America; 4 Center for Applied Social Research, University of Oklahoma, Norman, OK, United States of America; 5 Department of Civil, Construction, and Environmental Engineering, NC State University, Raleigh, NC, United States of America; 6 First Peoples Worldwide, University of Colorado, Boulder, CO, United States of America; 7 Robeson County Cooperative for Sustainable Development, Pembroke, NC, United States of America; 8 Department of Clinical Sciences as an Assistant Professor of Shellfish Pathology, NC State University, Morehead City, NC, United States of America; 9 Department of Marine Earth, and Atmospheric Sciences, NC State University, Raleigh, NC, United States of America

**Keywords:** geospatial analytics, coastal algae, public health, data curation, community engagement, social justice research

## 1. Introduction

In 1991, the National people of color summit introduced seventeen principles of environmental justice (EJ) to mobilize against exploitation of people and the environment [1]. The principles addressed widespread environmental injustices revealed through what was then a rare exchange: conversations between frontline communities and scientific ways of knowing.

Frontline communities experiencing environmental hazards ‘first and worst’ provided insight into how and why to integrate data sources. Advances in computing allowed for more sophisticated monitoring and data integration, highlighting systematic disparities in environmental conditions [1]. Divergence in knowledge revealed data abuse—or using data to deliberately co-locate hazards with communities of color, indigenous descent, and lower income [2, 3]. It was a breakthrough moment for EJ and a catalyst for future scientific and engineering conversations that foreground risk experiences not previously understood through scientific analyses.

**Figure 1.**
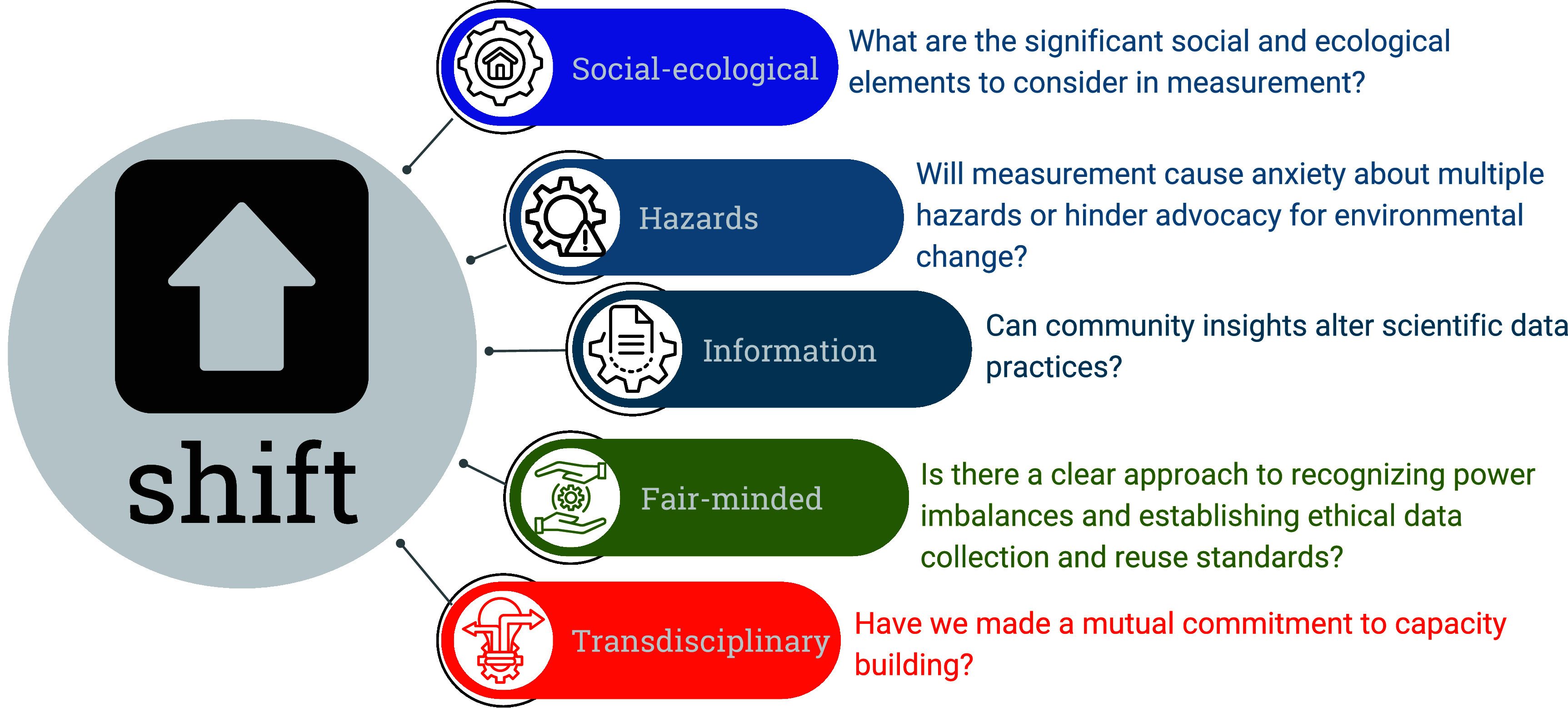
The social-ecological hazards information for fair transdisciplinarity framework (SHIFT framework).

Since then, advancements in generating and analyzing data on environmental injustice have surged, but sometimes fail to align with principles for EJ or collaboration [4] developed by EJ proponents. In this paper, we propose a strategy to address institutional and cultural barriers that may impede engineers and other environmental scientists from engaging in EJ work. Drawing on our experience and literature from EJ and engineering, we introduce the social-ecological hazards information for fair transdisciplinarity framework, abbreviated the SHIFT framework. This framework (figure 1) provides a playbook for the following processes:

• incorporating **social-ecological** context in measurements,
• evaluating stigma or fear of **hazards** during data collection,
• understanding **information** collection and data abuse,
• encouraging **fair-minded** practices to address power imbalances, and
• co-creating **transdisciplinary** knowledge for societal and scientific benefit.

The SHIFT framework emphasizes distributed leadership, connecting data to provenance, and participatory and transparent data care, thus avoiding the kind of abuse mentioned above. The SHIFT framework can subtly change the scholarly practice of historically and predominantly white and middle-class institutions whose actions have exploited, marginalized, and neglected the interests and environmental concerns of black, indigenous, and people of color (BIPOC) and other frontline communities. It also resists the misperception that deeper community engagement is always better.

Below, we review the SHIFT pilot project and a prospective application. The SHIFT pilot exemplified research amid EJ challenges such as floods and community health concerns. By collaborating across disciplines and respecting local knowledge, the project addressed data misalignment, misrepresentation, and missingness post-disaster. It highlighted how meaningful scientific and community outcomes require ethical data collection and interpretation [5]. The prospective case outlines how SHIFT reframed the community engagement approach proposed by a larger scale multi-investigator project funded by the National Institutes of Health and National Science Foundation (awards OCE-2414792 and 1P01ES035542-01). We describe how the SHIFT framework leads to a data democracy approach that reframes activities in ways that break assumptions that simply doing more science in the same ways will lead to better outcomes. This helps navigate a desire to appreciate and add value to communities that invest in research while attending to the institutional and disciplinary contexts that researchers must be trustworthy partners with the ability to support reforms in culture that enable the inclusive culture, critical thinking, and innovation that others have argued come through EJ and engineering e.g. [6, 7]. A positionality statement explains how institutional identity impelled this framework (supplemental material).

## 2. Piloting SHIFT: data misalignment, misunderstanding, and missingness after disaster

The pilot of the SHIFT framework took place in Robeson County, North Carolina, a rural and racially diverse area facing significant floods in 2016 and 2018, which caused extensive livestock and human waste contamination [8]. At the same time, residents of the region were organizing for indigenous sovereignty, resisting a fossil fuel pipeline, and expressing concerns about long-term exposure to drinking water contamination [9]. The complex riskscape and proximity to hazards is not uncommon in frontline communities.

In our case, pre-existing community relationships encouraged us to fill a data gap relevant to the community, but in a way that encouraged community leadership on the measurement timing and location, sampling location selection and data ownership. Social scientists and community members explained the value of the data to households and community leaders, including a community organizer and a team member with trauma counseling experience. We shared contact information to allow for on-going studies. Social scientists and community partners helped to steer interpersonal and inter-institutional politics such that it reflected our commitment to procedural fairness and shared leadership.

Engineers on the team helped explain regulatory thresholds and ensure scientifically sound results communication. At the same time, they embraced the generative opportunities of applying existing skills under different contexts: they could relate their stream water testing to soil contaminant protocols [8]. Although engineering peers sometimes raise questions about the ways community-driven sampling may bias results, we argue that the benefits to science (greater access to testing locations, community willingness to participate in science, the ability to test the assumptions of science when field locations are limited to public right of ways and other logistic barriers to sampling) are worthwhile and essential. The result was information with the potential to advance science and post-disaster recovery decisions in ways that shifted the conversation toward democracy: we deliberately avoided western notions about knowledge creation that may implicitly underpin choices and contribute to data misalignment, misrepresentation and misunderstanding in frontline communities.

**Misalignment** can occur when there’s a disconnect between the intended purpose of data collection and how it is ultimately used. Data collected for one specific purpose, like studying consumer preferences, might be repurposed to analyze voting patterns. While the data itself might be accurate, it may not capture the nuances needed to draw valid conclusions about voter behavior.

**Misrepresentation** arises when data is presented in a way that does not accurately reflect reality. Researchers present data that support their hypothesis without understanding outliers that may skew their results. This can produce misleading results and hinder our ability to make evidence-based decisions. For example, tools that focus on comparing nearby regions to one another to locate harm may overlook spatial aggregation. Misrepresentation is often the result of scaling issues, incomplete or missing data, or poor visualization.

**Missingness** refers to situations in which data is not collected at a fine enough resolution to capture important details or variations. This can lead to an underestimation of a phenomenon’s true complexity by hyper-fixating on a single scale, indicator of model validity, or mean condition. None of these measures of quality are without purpose, but when they get applied out of habit, problems arise.

The pilot demonstrated that scientists and analysts are fully capable of the mindset shifts that lead to EJ-informed decision making and data collection. It generated new applications of geospatial analytics to issues of disparity e.g. [10] and suggested that SHIFT could inform other projects that included engineering.

## 3. SHIFTing again: data democracy for harmful algal blooms (HABs)

To test the transferability of SHIFT to larger projects, The NC Center for Coastal Algae, People and the Environment used it to develop a community engagement strategy that centers fair procedures for understanding information sharing related to HABs. A full explanation of the environmental and social consequences of HABs is available in the literature [11, 12]. HABs are becoming more common across US waters and can lead to fishkills and diminished water quality. Their hepatotoxins, ingested via drinking water or contaminated seafood, raise additional concerns about an increase in non-alcoholic fatty liver disease [13]. Additional pollutants increase the ecological damage, and the stakes are high: commercial and recreational fishing account for $300 million in value, 5500 jobs, and a significant part of the region’s identity [14]. HABs themselves seem to be a relatively minor concern among frontline communities [12]. However, the ways that wealthier coastal communities adapt to changes in HABs, climate change, and other environmental concerns may deflect new risks to frontline communities [15].

Because frontline communities are often underserved, erased, or misrecognized by science—viewed, shortsightedly, as recipients of scientific advancements rather than contributors–we will prioritize two activities: researcher training in open and fair data standards and workshops engaging BIPOC environmental health leaders to develop a more expansive and relevant research and communication strategy.

The first activity—research training—is a responsibility of readiness to share power. By empowering researchers and academic trainees with the responsibility for change, we hope to realign the skills in data management and data publication already valued in many fields. This approach capitalizes on growing scientific interest in discovering how algorithmic biases misidentify where, how, and when environmental risks are highest; whose data are credible; and how to ensure access to and opportunities to scrutinize the data used in policy decisions [16, 17]. This is consistent with open data initiatives, such as The FAIR Principles, positing that data should be Findable, Accessible, Interoperable, and Reusable by other humans and machines. It also considers the ethics of rendering communities visible through data—both positive *and* negative [18]. The positive implications include recognition of environmental conditions that contribute to disease risk. The negative implications include the potential to be stigmatized and subjected to economic and ecological blackmail. Data generation, rights, and use are all elements of sovereignty separating data democracy from data authoritarianism. From a scientific standpoint, consideration of data needs, rights, and use provides a robust opportunity to question whether communities are overburdened by hazards or overburdened by hazard data that may be used to deem the community unworthy. The SHIFT framework encourages examination of procedures supporting democratization, engagement, and autonomy [19].

The second investment in data democracy realized through the SHIFT framework is that we can gain trust and improve science by testing our information sharing approaches in EJ convenings first. In North Carolina, there are at least three networks of frontline communities that convene organizations aiming to promote health and environmental equality for all people, community action for environmental conservation, clean industry, and safe workplaces. For example, the NC EJ Summit began convening BIPOC environmental health leaders in the 1990s. Today, over 27 partner organizations are meeting to discuss common challenges, data needs, and strategies for civic engagement. Many come with the expectation of deliberating research and sharing ideas with university research teams. By registering for the summit and paying the institutional registration fee, we contribute to EJ leadership without overextending our institutional and disciplinary resources. The environmental health leaders that convene at NC EJ Network Summits are familiar with the technical and educational needs of their communities. This makes them uniquely positioned to co-develop and scrutinize our interpretations of the benefits of research and identify common ethical blunders that may undermine the relevance of data to their work and communities. In contrast to hosting our own HABs-specific meeting, this approach situates the team’s focus within community goals, de-centering our own needs.

## 4. Conclusion

The practices identified through SHIFT can leverage high-level knowledge sharing across disciplinary boundaries while honoring the resources, knowledge, and accountability systems that partners bring to collaboration [20]. As such, the SHIFT framework aligns EJ with commitment to the engineering principle of data democracy—that is, consideration of where, how, and with whom engineers and other academic colleagues generate, use, and translate data for social good. It also considers how they identify and contest data abuse, misalignment, misunderstanding, and missingness.

The goal of the SHIFT framework is to identify strategies that reform academic and disciplinary institutional cultures while recognizing that to adopt the status quo is to perpetuate scientific ideals that are complicit in exclusion and exploitation. The SHIFT framework is a sort of field guide for approaching those changes—even (or perhaps especially) for scientists and engineers who are wary of their personal capacity to participate in EJ-adjacent research or who perceive institutional or professional barriers to success. Often, it is these very technical experts who know best how to measure, map, and project the impacts of EJ concerns. By moving responsibility for reform toward data practices, the SHIFT framework intends to help make strategic decisions about how to do EJ work with frontline communities instead of to communities.

## Data Availability

No new data were created or analysed in this study.
